# Very-Short-Term Sleep Deprivation Slows Early Recovery of Lymphocytes in Septic Patients

**DOI:** 10.3389/fmed.2021.656615

**Published:** 2021-05-24

**Authors:** Yujing Zhang, Yuming Wu, Dan Xu, Ping Xiao, Bing Xie, Haiyan Huang, You Shang, Shiying Yuan, Jiancheng Zhang

**Affiliations:** ^1^Department of Critical Care Medicine, Tongji Medical College, Union Hospital, Huazhong University of Science and Technology, Wuhan, China; ^2^Tongji Medical College, Institute of Anesthesia and Critical Care Medicine, Union Hospital, Huazhong University of Science and Technology, Wuhan, China

**Keywords:** atrial natriuretic peptide, intensive care unit, lymphocytes, RCSQ, short-term sleep deprovation

## Abstract

Sleep plays an important role in immune function. However, the effects of very-short-term sleep deprivation on the early recovery of immune function after sepsis remain unclear. This study was conducted in the intensive care unit to investigate the effects of 2 consecutive days of sleep deprivation (SD) on lymphocyte recovery over the following few days in septic patients who were recovering from a critical illness. The patients' self-reports of sleep quality was assessed using the Richards–Campbell Sleep Questionnaire at 0 and 24 h after inclusion. The demographic, clinical, laboratory, treatment, and outcome data were collected and compared between the good sleep group and poor sleep group. We found that 2 consecutive days of SD decreased the absolute lymphocyte count (ALC) and ALC recovery at 3 days after SD. Furthermore, post-septic poor sleep decreased the plasma levels of atrial natriuretic peptide (ANP) immediately after 2 consecutive days of SD. The ANP levels at 24 h after inclusion were positively correlated with ALC recovery, the number of CD3^+^ T cells, or the number of CD3^+^ CD4^+^ cells in the peripheral blood on day 5 after inclusion. Our data suggested that very-short-term poor sleep quality could slow down lymphocyte recovery over the following few days in septic patients who were recovering from a critical illness. Our results underscore the significance of very-short-term SD on serious negative effects on the immune function. Therefore, it is suggested that continuous SD or several short-term SD with short intervals should be avoided in septic patients.

## Introduction

Sleep and immunologic function are bidirectionally related (1). Sleep is a fundamental physiologic process required for facilitation of the significant restorative processes and plays important roles in the balanced homeostatic regulation of the immune system (1–3). Disturbed sleep and circadian rhythm disruption impair systemic immunological responses including innate and adaptive immune responses and activate inflammation with increased circulating inflammatory cytokines (4–6). Sleep deprivation abrogates the circadian rhythm of the number and function of CD4^+^ CD25^+^ natural regulatory T cells and dampens CD4^+^ CD25^−^ T cell proliferation in the peripheral blood (7). Evidence have shown that sleep plays important roles in fine-tuning the immune system to foster immune defense and help fight infections (8). Sleep rhythm disturbance, shorter sleep duration, or poor sleep quality could weaken our body's defense system and thus render the body more prone to various infections caused by different pathogens, including viruses and bacteria (9–13). Evolutionary increases in mammalian sleep durations are strongly associated with a higher number of circulating immune cells and substantially reduced the levels of parasitic infection (14). Our previous study found that self-reported poor sleep quality during hospitalization in patients with corona virus disease 2019 (COVID-19) is associated with slow recovery from lymphopenia and prolonged duration of hospital stay (15). Taken together, sleep and the circadian rhythm system exert a strong regulatory influence on immune homeostasis and immune defense.

Sleep disruption is a highly prevalent problem in the intensive care unit (ICU) due to many potential sleep disruptions, including environmental factors (noise, continuous light exposure, frequent patient care activities, mechanical ventilation, medications, *etc*.) and patient-related factors (underlying illness, the pathophysiology of the acute illness, pain, psychological factors, *etc*.) (16, 17). Sepsis caused by infection is one of the most common causes of morbidity and mortality in the ICU (18). There are bidirectional crosstalks between infection with sleep impairment (1). Substantial evidence supporting the view that infection *per se* can lead to sleep disturbances has been accumulated (19–22). Conversely, septic patients or septic mice that underwent frequent sleep disruptions have higher mortality rates (23, 24). Both clinical and animal studies showed that short-term acute sleep loss for up to 2 days could transiently dampen natural killer cell activity in the blood (25–28) and inhibit lymphocyte proliferation (7, 29). However, there are currently no reports of the effects of very-short-term sleep quality on the absolute counts of peripheral blood lymphocytes and the recovery of lymphocytes over the following few days in septic ICU patients. In our study, we aimed to prospectively compare the recovery of lymphocytes and clinical outcomes in septic ICU patients with 2 consecutive days of self-reported good or poor sleep. We focused on the investigation that even very-short sleep deprivation in septic patients could have a significant negative impact on the recovery of immune function over the following few days. The plasma atrial natriuretic peptide (ANP) levels were also detected two times immediately after each day's sleep assessment to elucidate the possible mechanisms of the effects of short-term sleep deprivation on the recovery of lymphocyte in septic ICU patients.

## Materials and Methods

### Study Design and Participants

**Figure 1:**
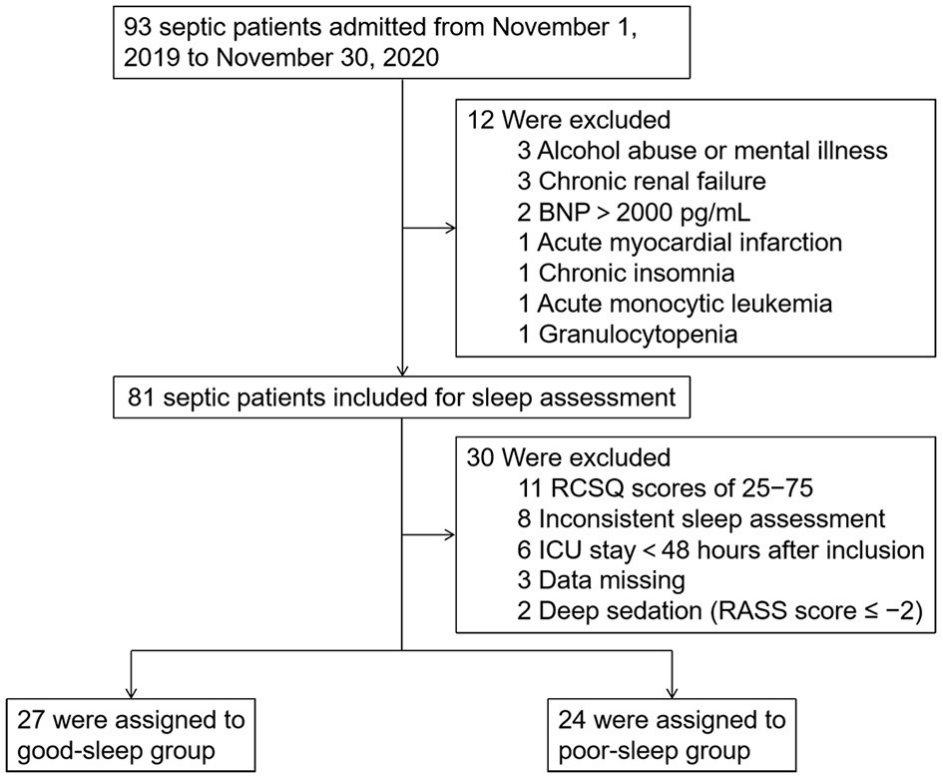
Study flow diagram. BNP, brain natriuretic peptide; ICU, intensive care unit; RCSQ, Richards–Campbell Sleep Questionnaire.

For this single-center prospective observational study, 93 septic patients admitted to the integrated ICU of Wuhan Union Hospital between November 1, 2019 and November 30, 2020 were enrolled after meeting the inclusion criteria, which are as follows: (1) patients aged between 18 and 80 years, (2) sequential organ failure assessment (SOFA) score ≥ 2, (3) neurological status allowing communication (alert, oriented, responding to commands), and (4) no visual or hearing impairment. The exclusion criteria included the following: (1) delirium was present during the study period, which was assessed twice daily by trained ICU nurses using the Confusion Assessment Method for the ICU (CAM-ICU), (2) ICU length of stay >30 days at the time of inclusion, (3) acute myocardial infarction or brain natriuretic peptide (BNP) >2,000 pg/ml, (4) alcohol abuse or mental illness, (5) chronic renal failure, (6) chronic insomnia, (7) pregnant or lactating women, and (8) autoimmune disease or hematological malignancy. A total of 81 patients met these criteria and were included in the study (Figure 1).

All invasive mechanically ventilated patients were mainly sedated with propofol with an intravenous pump before and after inclusion when needed.

The study was approved by the Ethics Committee of Union Hospital, Tongji Medical College, Huazhong University of Science and Technology (permission number: S1164) and registered on the Chinese Clinical Trial Registry (ChiCTR) site (http://www.chictr.org.cn; registration number: ChiCTR1900025497). Informed consent was obtained prior to study enrolment.

### Sleep Assessment

At 0 and 24 h after inclusion, self-reported sleep quality was evaluated between 8:00 and 9:00 a.m. using the Richards-Campbell Sleep Questionnaire (RCSQ), a widely used subjective survey instrument in the ICU (30–32). The RCSQ is a validated five-item questionnaire on a 0–100-mm visual analog scale to evaluate perceived sleep depth, sleep latency, number of awakenings, latency after awakenings, and sleep quality. Higher RCSQ scores indicate better sleep quality, and the average value of these five items represent overall sleep quality. Self-reported factors associated with disruptive sleep were also recorded. After the patients' self-reported sleep assessment, we further checked the patients' sleep quality by asking the night-shift nurses who completed the RCSQ regarding their patients' overnight sleep quality.

During the study period, 30 out of the 81 patients included were further excluded as 11 patients with RCSQ scores of 25–75, eight patients with inconsistent sleep assessment at 0 and 24 h after inclusion, six patients with length of ICU stay <48 h after inclusion, three patients with clinical and laboratory data missing, and two patients who received deep sedation (Richmond Agitation–Sedation Scale score during the study period ≤-2). The 51 patients eventually included were divided into two groups according to the RCSQ scores: good sleep group (two RCSQ scores ≥75) and poor sleep group (two RCSQ scores ≤25).

### Sleep-Promoting Interventions

At 24 h after inclusion, earplugs and eye masks were used at night to help poor sleep patients sleep better. If the patients in the good sleep group reported poor sleep 24 h after inclusion, earplugs and eye masks were also used at night as a sleep aid. RCSQ was assessed at 48, 72, 96, and 120 h after inclusion.

While earplugs and eye masks were available for clinicians to employ at their clinical discretion, the use of other sleep improvement strategies was not protocolized during the study period.

### Data Collection

Basic demographic and clinical data were collected at the time of inclusion: age, sex, body mass index, marital status, education level, comorbidities (cardiovascular and cerebrovascular diseases, endocrine disease, malignancy, chronic respiratory disease, and gastrointestinal disease), length of ICU stay before inclusion (hours), mechanical ventilation time before inclusion (hours), days of analgesia and sedation before inclusion, and treatment (invasive mechanical ventilation, antibiotics, vasoconstrictive agents, immunoregulatory therapy, corticosteroids, immunoglobulin). Data on laboratory parameters [blood routine, blood biochemistry, cardiac biomarkers (troponin I, TnI, and creatine kinase-MB, CK-MB), BNP] were collected at −24, 0, and 24 h after inclusion, which were measured in the laboratory of the Wuhan Union Hospital. The blood routine test (white blood cell count, neutrophil count, lymphocyte count, platelet count, *etc*.) was performed using BC-3000 auto-hematology analyzer (Mindray, Shenzhen, China). Blood biochemistry (including indices of renal and hepatic functions) was tested using BS-200 automatic biochemical analyzer (Mindray, Shenzhen, China). The level of CK-MB was analyzed using a commercially available kit (eBioscience, An Affymetrix Company, San Diego, CA, USA), and TnI was detected using a TnI assay kit (Cobas, Roche Diagnostics, USA). BNP was determined by CMIA using the ARCHITECT i2000 System and ARCHITECT BNP Reagent Kits (Abbott Laboratories, IL, USA). The plasma ANP levels of the patients were detected by enzyme-linked immunosorbent assay (ELISA) in the laboratory of the Institute of Anesthesia and Critical Care Medicine at 0 and 24 h after inclusion. The Acute Physiology and Chronic Health Evaluation (APACHE) II score and SOFA score were assessed at 0 and 24 h after inclusion. The absolute counts of peripheral blood lymphocytes were recorded on days 3, 4, and 5 after inclusion. All data were checked by two researchers.

### Outcomes

The primary outcome was the recovery rate based on absolute lymphocyte count (ALC) on days 3, 4, and 5 after inclusion. The absolute counts of peripheral blood lymphocytes at −24 h after inclusion were set as the baseline value. The formula for the recovery percentage is described as: $\frac{\text{ALC }\left(\text{day }3,\text{ day }4\text{ or day }5\right)\;-\text{ALC }\left(\text{baseline}\right)}{\text{ALC }\left(\text{baseline}\right)}\times 100\%.$

The secondary outcomes were as follows: (1) ALC on days 3, 4, and 5 after inclusion and (2) total length of ICU stay.

### ELISA Detection of ANP

At 0 and 24 h after inclusion, blood samples from each patient were collected and immediately centrifuged at 2,000 *g* for 15 min, and then the plasma aliquots were separated and stored at −80°C until analyses. Commercial ELISA kits (RayBiotech, Inc., Norcross, GA; Cat#: EIAM-ANP) were used following the kit protocols to determine the plasma ANP levels. After the ELISA procedure has been performed, the microtiter plate is read on an ELISA reader (Elx800, Bio-Tek Instrument Inc., USA) at a wavelength of 450 nm. The results were expressed in pg/ml, taking the given sensitivity values into consideration and calculating from the standard curve.

### Flow Cytometry Assay

To measure the lymphocyte subsets, 100 μl of whole peripheral blood was incubated in 900 μl of Tris-NH4Cl potassium lysis buffer (Thermo Fisher Scientific, Waltham, MA) at room temperature for 5 min to lyse the red blood cells. After two times of washing with phosphate-buffered saline, the lymphocyte subsets were measured with a BD Multitest™ 6-color TBNK reagent (BD Biosciences, San Jose, CA) according to the manufacturer's instruction.

### Statistical Analysis

Categorical variables were described as number (%). Proportions for categorical variables were compared using the χ^2^ test, Yates' continuity corrected χ^2^ test, or Fisher's exact test. Continuous variables were tested for normal distribution using the Kolmogorov–Smirnov test and Shapiro–Wilk test. Continuous variables were described using mean (SD) if they were normally distributed or median (interquartile range, IQR) if they were not. Continuous variables were compared using independent group *t*-tests when the data were normally distributed; otherwise, Mann–Whitney test was used. Correlation was determined by Pearson correlations. A two-sided *P* < 0.05 was considered statistically significant. The data collected were all analyzed using SPSS, version 20.0, software (SPSS, Tokyo, Japan).

## Results

### Patients

Overall, 93 septic patients admitted to the integrated ICU of Wuhan Union Hospital between November 1, 2019 and November 30, 2020 were included in the study. Subsequently, we excluded the following patients: 11 patients with RCSQ scores of 25–75, eight patients with two inconsistent sleep assessment, six patients with length of ICU stay <48 h after inclusion, three patients with alcohol abuse or mental illness, three patients with chronic renal failure, three patients with clinical and laboratory data missing, two patients with BNP >2,000 pg/ml, two patients who received deep sedation, one patient with acute myocardial infarction, one patient with chronic insomnia, one patient with acute monocytic leukemia, and one patient with granulocytopenia. Therefore, we included 51 patients (27 in the good sleep group and 24 in the poor sleep group) in the final analysis (Figure 1).

### Self-Reported Sleep Quality

Patients in the poor sleep group had lower RCSQ scores at the time of inclusion [median, 20 (IQR, 10–23) *vs*. 80 (IQR, 78–88), *P* < 0.0001] and 24 h after inclusion [median, 19 (IQR, 14–20) *vs*. 80 (IQR, 77–90), *P* < 0.0001] than those in the good sleep group (Table 1). Included among the etiological causes of poor sleep are environmental factors, psychological factors, and discomfort caused by an illness for 83.3, 62.5, and 33.3% of patients, respectively (Table 1).

**Table 1:** Sleep quality assessment in septic patients.

	**Median (IQR)**		***P*-value**
	**Good sleep (*n =* 27)**	**Poor sleep (*n =* 24)**	
RCSQ scores at the time of inclusion, median (IQR)	80 (78–88)	20 (10–23)	<0.0001^a^
RCSQ scores 24 h after inclusion, median (IQR)	80 (77–90)	19 (14–20)	<0.0001^a^
Factors affecting sleep quality			
Environmental factor, n (%)	–	20 (83.3)	–
Psychological factor, n (%)^a^	–	15 (62.5)	–
Discomfort caused by the illness, n (%)	–	8 (33.3)	–
RCSQ scores 48 h after inclusion, median (IQR)	78 (67–83)	68 (60–82)	0.0814
RCSQ scores 72 h after inclusion, median (IQR)	70 (55–78)	70 (60–80)	0.4497
RCSQ scores 96 h after inclusion, median (IQR)	75 (60–86)	66 (55–80)	0.1392
RCSQ scores 120 h after inclusion, median (IQR)	80 (60–85)	70 (54–84)	0.2983

*Values are median [IQR [range]] unless stated otherwise*.

a *Psychological factors include fear, anxiety, helplessness, and depression. IQR, interquartile range; n, number; RCSQ, Richards Campbell sleep questionnaire. P-values indicate differences between the good-sleep and the poor-sleep patients*.

a *P < 0.05 was considered statistically significant*.

There were no significant differences in the RCSQ scores between previous poor sleep patients and previous good sleep patients at 48, 72, 96, and 120 h after inclusion (Table 1; all *P* > 0.05).

### Basic Demographic and Clinical Data, Received Treatment at the Time of Inclusion, and Laboratory Parameters at −24, 0, and 24 h After Inclusion

The median age of the patients was 58 years (IQR, 46–68 years), 34% of them were men, and the median body mass index of patients was 23.7 kg/m^2^ (IQR, 20.3–25.7 kg/m^2^) (Table 2). There were no significant between-group differences in the basic demographic and clinical data as well as the received treatment at the time of inclusion (Table 2). However, the patients in the poor sleep group received longer days of sedation before inclusion (Table 2).

**Table 2:** Demographics and baseline characteristics of septic patients.

	**Total (*n =* 51)**	**Good sleep (*n =* 27)**	**Poor sleep (*n =* 24)**	***P*-value**
Age, median (IQR), years	58 (46–68)	60 (44–67)	58 (47–69)	0.7468
Sex				
Male	34 (66.7)	18 (66.7)	16 (66.7)	>0.9999
Female	17 (33.3)	9 (33.3)	8 (33.3)	>0.9999
BMI, median (IQR), kg/m^2^	23.7 (20.3–25.7)	23.7 (20.7–26.0)	23.0 (19.2–25.7)	0.7187
Marital status				
Married	48 (94.1)	26 (96.3)	22 (91.7)	0.4831
Unmarried	2 (39.2)	1 (3.7)	1 (4.2)	0.5237
Death of a spouse	1 (19.6)	0 (0)	1 (4.2)	0.4706
Education level				
≤ High school	48 (94.1)	25 (92.6)	23 (95.8)	0.6235
University or college	3 (5.9)	2 (7.4)	1 (4.2)	0.9162
Comorbidities				
Cardiovascular and cerebrovascular diseases	16 (31.4)	7 (25.9)	9 (37.5)	0.3739
Endocrine disease	1 (2.0)	0 (0)	1 (4.2)	0.4706
Malignancy	5 (9.8)	1 (3.7)	4 (16.7)	0.2792
Chronic respiratory disease	4 (7.8)	2 (7.4)	2 (8.3)	0.6899
Gastrointestinal disease	7 (13.7)	4 (14.8)	3 (12.5)	0.8667
Length of ICU stay before inclusion (hours)	111 (47–216)	72 (48–216)	120 (31–251)	0.9292
Mechanical ventilation time before inclusion (hours)	48 (0–158)	48 (0–144)	58 (0–297)	0.4628
Days of sedation before inclusion (days)	2.0 (1.0–5.0)	1.0 (0.0–3.0)	4.5 (1.0–7.0)	0.0029^a^
Days of analgesia before inclusion (days)	3.0 (1.0–5.0)	2.0 (0.0–5.0)	4.0 (1.0–6.0)	0.2190
Treatment at inclusion				
Invasive mechanical ventilation	16 (31.4)	7 (25.9)	9 (37.5)	0.3739
Antibiotics	51 (100)	27 (100)	24 (100)	–
Vasoconstrictive agents	5 (9.8)	1 (3.7)	4 (16.7)	0.2792
Immunoregulatory therapy	0 (0)	0 (0)	0 (0)	–
Corticosteroids	0 (0)	0 (0)	0 (0)	–
Immunoglobulin	0 (0)	0 (0)	0 (0)	–

*Values are numbers (percentages) unless stated otherwise. IQR, interquartile range; n, number. P-values indicate differences between good-sleep and poor-sleep patients*.

a *P < 0.05 was considered statistically significant*.

No significant between-group differences were detected in the laboratory parameters at −24, 0, and 24 h after inclusion, including white blood count, neutrophil count, monocyte count, lymphocyte count, platelet count, hemoglobin, total bilirubin, blood urea nitrogen, serum creatinine, creatine kinase-MB, hypersensitive cardiac troponin I, and BNP (Table 3). There was also no significant between-group difference in the APACHE II score and SOFA score at 0 and 24 h after inclusion (Table 3). No significant between-group difference in total length of ICU stay was observed (Table 3).

**Table 3:** Laboratory parameters at −24, 0 and 24 h after inclusion, and total length of ICU stay.

	**Normal range**	**Hours after inclusion**	**Total (*n =* 51)**	**Good-sleep (*n =* 27)**	**Poor-sleep (*n =* 24)**	***P*-value**
**Blood routine**						
White blood count, ×10^9^/L	3.50–9.50	−24	10.53 ± 5.19	10.41 ± 5.06	10.66 ± 5.44	0.8672
		0	10.51 ± 4.57	9.79 ± 3.94	11.31 ± 5.16	0.2411
		24	10.08 ± 4.22	9.88 ± 3.80	10.31 ± 4.71	0.7199
Neutrophil count, ×10^9^/L	1.80–6.30	−24	7.71 (5.70–11.52)	7.86 (4.94–9.93)	7.08 (6.14–11.94)	0.8169
		0	8.80 ± 4.34	8.15 ± 3.74	9.52 ± 4.91	0.2652
		24	8.55 ± 4.28	8.39 ± 4.14	8.73 ± 4.51	0.7746
Monocyte count, ×10^9^/L	0.10–0.60	−24	0.50 (0.36–0.81)	0.59 (0.36–0.87)	0.47 (0.36–0.60)	0.5526
		0	0.54 (0.42–0.88)	0.62 (0.45–0.96)	0.49 (0.39–0.85)	0.4015
		24	0.53 (0.37–0.82)	0.59 (0.49–0.88)	0.48 (0.23–0.74)	0.0781
Lymphocyte count, ×10^9^/L	1.10–3.20	−24	0.78 (0.52–1.15)	0.80 (0.41–0.95)	0.73 (0.53–1.36)	0.8363
		0	0.74 (0.57–1.26)	0.70 (0.57–1.14)	0.84 (0.57–1.42)	0.5151
		24	0.86 (0.57–1.16)	0.83 (0.58–1.33)	0.87 (0.44–1.14)	0.5712
Platelet count, ×10^9^/L	125–350	−24	154 (67–224)	160 (68–246)	154 (139–214)	0.4929
		0	189 ± 114	202 ± 127	173 ± 98	0.3723
		24	204 ± 116	217 ± 131	190 ± 96	0.4053
Hemoglobin, g/L	130–175	−24	86 ± 19	86 ± 19	86 ± 20	0.9912
		0	86 ± 19	86 ± 17	87 ± 21	0.9280
		24	83 (70-100)	83 (75-100)	83 (67-100)	0.5967
**Blood biochemistry**						
Albumin, g/L	33.0–55.0	−24	27.9 (26.2–31.3)	28.0 (26.2–32.3)	27.4 (25.4–30.7)	0.8260
		0	28.6 (26.2–31.3)	28.9 (27.0–31.7)	27.7 (25.5–30.6)	0.4340
		24	28.5 (25.9–31.0)	29.4 (27.2–32.0)	26.0 (25.4–29.3)	0.0138^a^
Total bilirubin, μmol/L	3.0–20.0	−24	15.9 (8.7–28.4)	17.6 (7.8–34.0)	15.4 (8.8–27.7)	0.9674
		0	17.6 (8.1–34.5)	19.8 (8.1–35.5)	14.4 (8.1–30.9)	0.3460
		24	15.7 (8.3–31.6)	16.6 (10.9–50.7)	12.1 (7.9–25.1)	0.4396
Blood urea nitrogen, mmol/L	2.90–8.20	−24	7.32 (5.07–12.43)	6.83 (4.19–12.60)	7.91 (5.50–11.16)	0.6009
		0	8.60 (6.02–10.86)	7.52 (4.87–12.43)	8.75 (6.82–10.26)	0.7898
		24	7.95 (6.00–11.68)	7.69 (4.78–13.32)	8.38 (6.01–9.89)	0.9181
Serum creatinine, μmol/L	57.0–111.0	−24	55.1 (41.7–90.3)	59.4 (46.3–102.6)	52.2 (34.8–93.6)	0.2688
		0	51.2 (40.8–84.9)	51.2 (42.3–92.4)	51.0 (32.8–84.9)	0.3732
		24	52.2 (39.3–80.6)	58.9 (41.6–90.0)	49.3 (32.2–78.7)	0.2203
**Cardiac biomarkers**^**†**^						
Creatine kinase-MB ≥ 6.6, ng/mL	<6.6	−24	3 (5.9)	1 (3.7)	2 (8.3)	0.9162
		0	4 (7.8)	2 (3.5)	2 (8.3)	0.6899
		24	1 (2.0)	0 (0)	1 (4.2)	0.4706
Hypersensitive cardiac troponin I ≥ 26.2, ng/L	<26.2	−24	10 (19.6)	5 (18.5)	5 (20.8)	0.8354
		0	14 (27.5)	7 (2.6)	7 (2.9)	0.7957
		24	10 (19.6)	5 (18.5)	5 (20.8)	0.8354
BNP^‡^, pg/mL	<100	−24	227.5 (91.5–513.7)	227.5 (55.7–407.2)	235.9 (93.4–869.8)	0.5102
		0	122.9 (53.2–357.1)	121.7 (44.4–416.6)	122.9 (59.7–215.0)	0.8665
		24	114.8 (31.8–517.3)	101.7 (27.2–715.6)	123.9 (33.7–350.9)	0.9349
ANP, pg/mL	–^§^	0	31.03 (23.43–50.09)	37.30 (25.69–82.83)	26.41 (17.84–40.46)	0.0775
		24	37.40 (25.35–55.55)	45.94 (36.05–61.54)	30.48 (15.30–43.72)	0.0079^a^
APACHE II score	–	0	11 (9–13)	10 (8–12)	13 (9–14)	0.0691
		24	11 (7–13)	11 (6–13)	11 (8–14)	0.2559
SOFA score	–	0	3 (2–4)	3 (2–4)	4 (2–4)	0.6776
		24	3 (2–5)	4 (2–5)	3 (2–5)	0.6217
Total length of ICU stay, median (IQR), d	–	–	9.0 (4.0–15.0)	9.0 (4.0–16.0)	8.5 (6.0–15.0)	0.7108

*Values are mean ± standard deviation (SD), median [IQR [range]] or numbers (percentages). APACHE II, acute physiology and chronic health evaluation II; IQR, interquartile range; n, number; SOFA, sequential organ failure assessment*.

a *P-values indicate differences between good-sleep and poor-sleep patients*.

a *P < 0.05 was considered statistically significant*.

† *Data regarding cardiac biomarkers at 24 h pior to inclusion were missing for 12 patients (44.4%) in the good-sleep group, and 13 patients (54.2%) in the poor-sleep group*.

‡ *Data were missing for the measurement of BNP at 24 h pior to inclusion in 10 patient (37.0%) in the good-sleep gqroup, and eight patients (33.3%) in the poor-sleep group*.

§ *The plasma ANP levels of seven healthy adult volunteers were 0.64 pg/mL (IQR, 0.60–3.69 pg/mL)*.

However, the blood albumin level was significantly lower in the poor sleep group compared to those in the good sleep group at 24 h after inclusion [median, 26.0 g/L (IQR, 25.4–29.3 g/L) *vs*. 29.4 g/L (IQR, 27.2–32.0 g/L), *P* = 0.0138], but not at −24 and 0 h after inclusion (Table 3).

### Plasma ANP Levels at 0 and 24 h After Inclusion

Patients in the poor sleep group had a decreased plasma level of ANP compared to those in the good sleep group at 24 h after inclusion [median, 31.28 pg/ml (IQR, 15.44–47.49 pg/mL) *vs*. 45.94 pg/ml (IQR, 36.05–61.54 pg/ml), *P* = 0.0138], but not at the time of inclusion (*P* = 0.0775) (Table 3).

### The ALC Recovery on Days 3, 4, and 5 After Inclusion

**Figure 2:**
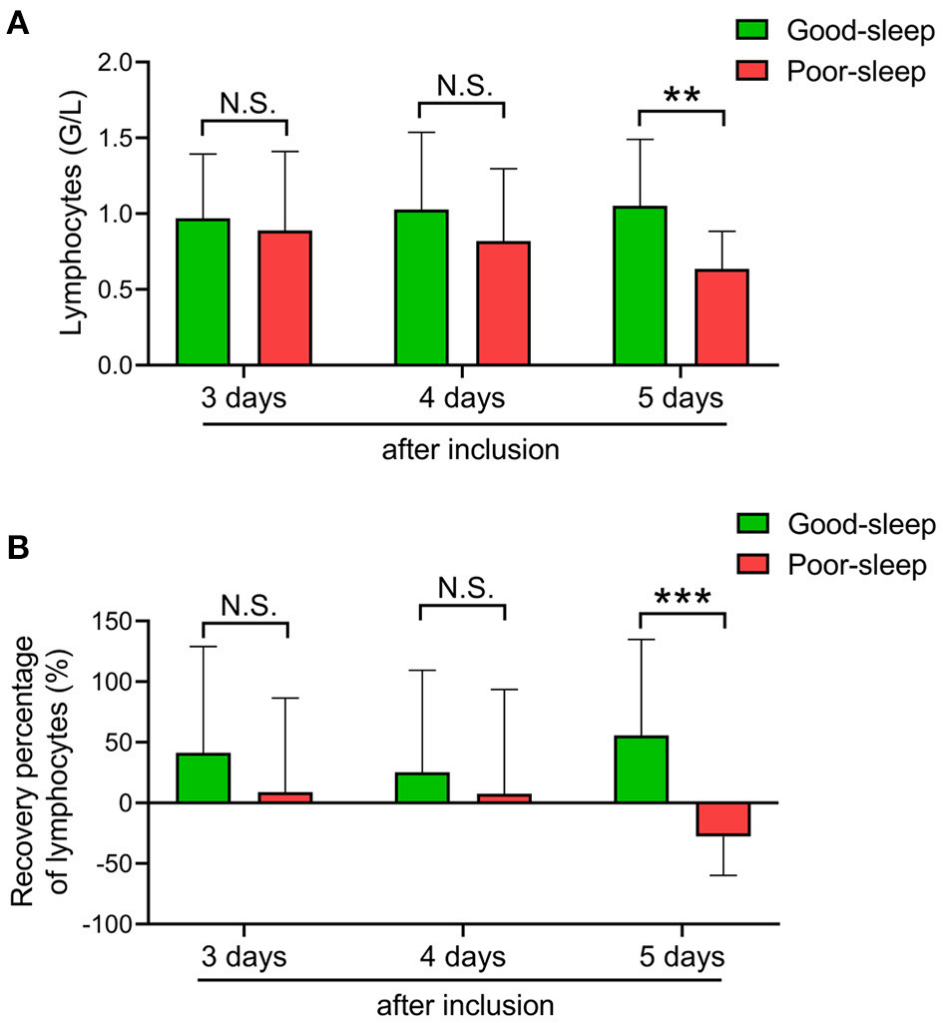
Effects of short-term sleep deprivation on the recovery of absolute lymphocyte count (ALC) in septic intensive care unit patients. **(A)** Dynamic changes in ALC at 3, 4, and 5 days after inclusion in septic patients with good or poor sleep. **(B)** Dynamic changes in ALC recovery rate at 3, 4, and 5 days after inclusion in septic patients with good or poor sleep. Data are shown as mean ± SD. **P < 0.01 and ***P < 0.001. N.S., not significant.

Compared to patients in the good sleep group, patients in the poor sleep group had significantly decreased ALC on day 5 after inclusion [median, 0.62 × 10^9^/L (IQR, 0.39–0.85 × 10^9^/L) *vs*. 0.87 × 10^9^/L (IQR, 0.72–1.38 × 10^9^/L), *P* = 0.0017], but not on days 3 and 4 after inclusion (Figure 2).

The recovery rate of ALC was also higher in patients with good sleep than those with poor sleep on day 5 after inclusion [median, 47.4% (IQR, 9.95–105.18%) *vs*. −25.00% (IQR, –41.79–3.85%), *P* = 0.0006], but not on days 3 and 4 after inclusion (Figure 2).

### Correlation Coefficient Analysis of Plasma ANP Levels With ALC, ALC Recovery, or Lymphocyte Subsets in the Peripheral Blood

**Figure 3:**
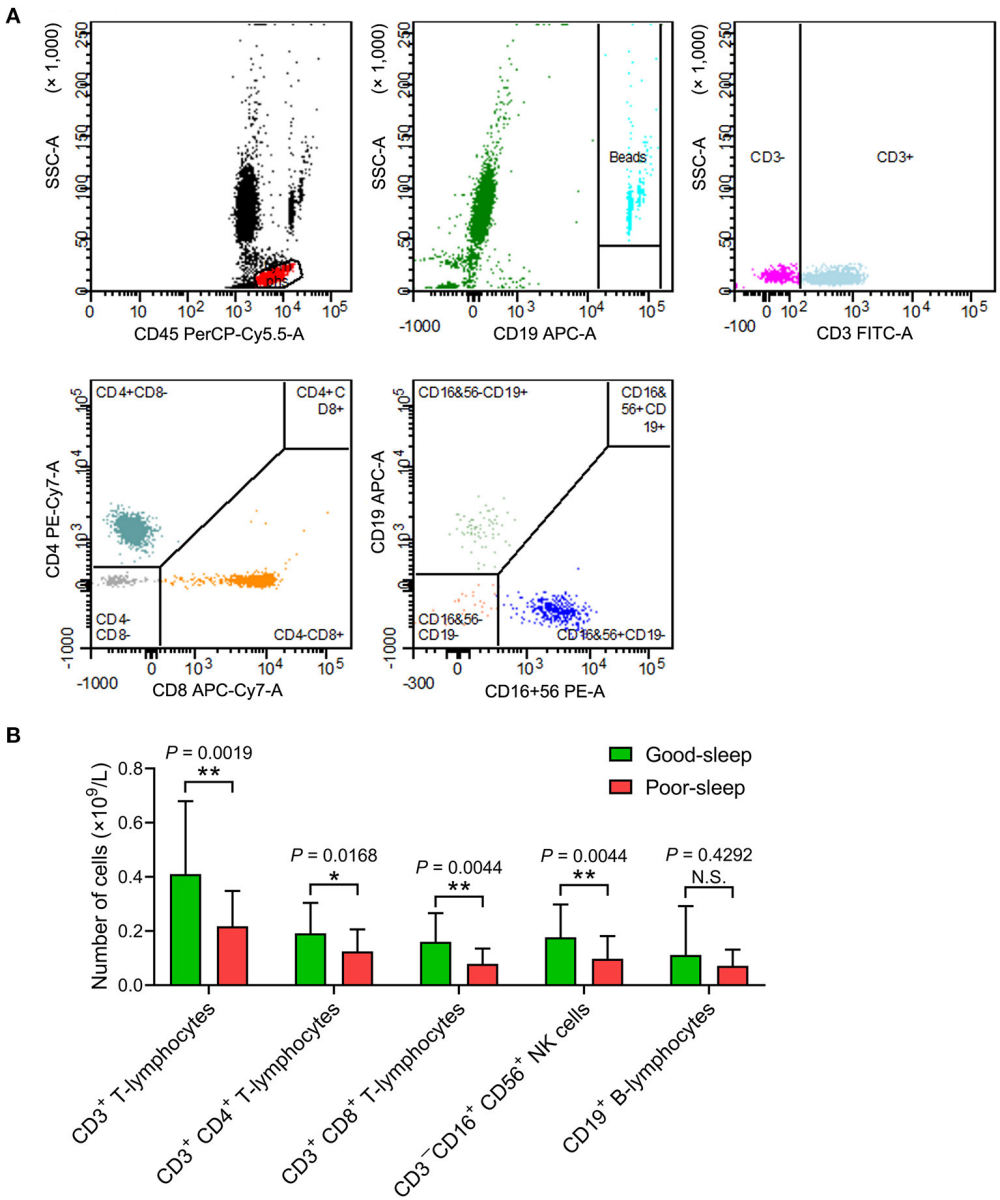
Effects of post-septic sleep deprivation on the number of lymphocyte subsets. **(A,B)** Flow cytometry analysis of CD3^+^ T-lymphocytes, CD3^+^ CD4^+^ T-lymphocytes, CD3^+^ CD8^+^ T-lymphocytes, CD3^−^CD16^+^ CD56^+^ NK cells, and CD19^+^ B-lymphocytes in peripheral blood on day 5 after inclusion. Data are shown as mean ± SD. *P < 0.05 and **P < 0.01. N.S., not significant; NK cells, natural killer cells.

The septic patients with poor sleep had a lower number of CD3^+^ T-lymphocytes, CD3^+^ CD4^+^ T-lymphocytes, CD3^+^ CD8^+^ T-lymphocytes, and CD3^−^CD16^+^ CD56^+^ natural killer (NK) cells in the peripheral blood compared to septic patients with good sleep on day 5 after inclusion (Figure 3).

In all patients, correlation coefficient analysis showed a positive correlation between plasma ANP levels at 24 h after inclusion and ALC recovery on day 5 after inclusion (*r* = 0.394, *P* = 0.018) (Table 4). The plasma ANP levels at 24 h after inclusion also showed a positive correlation with ALC recovery on day 5 after inclusion among the patients with good sleep (*r* = 0.470, *P* = 0.027) or the patients with poor sleep (*r* = 0.527, *P* = 0.044) (Table 4).

**Table 4:** Correlation coefficient analysis of plasma ANP levels at 24 h after inclusion with ALC on day 5 after inclusion and recovery of ALC on day 5 after inclusion.

	**ALC on day 5 after inclusion**			**Recovery of ALC on day 5 after inclusion**		
	**Total**	**Good-sleep**	**Poor-sleep**	**Total**	**Good-sleep**	**Poor-sleep**
Plasma ANP levels at 24 h after inclusion	*r =* 0.192 *P* = 0.262	*r =* 0.130 *P* = 0.565	*r =* 0.168 *P* = 0.567	*r =* 0.394^*^ P = 0.018	*r =* 0.470^*^ P = 0.027	*r =* 0.527^*^ P = 0.044

*ALC, absolute lymphocyte count; ANP, atrial natriuretic peptide*.

* *P < 0.05 was considered statistically significant*.

**Figure 4:**
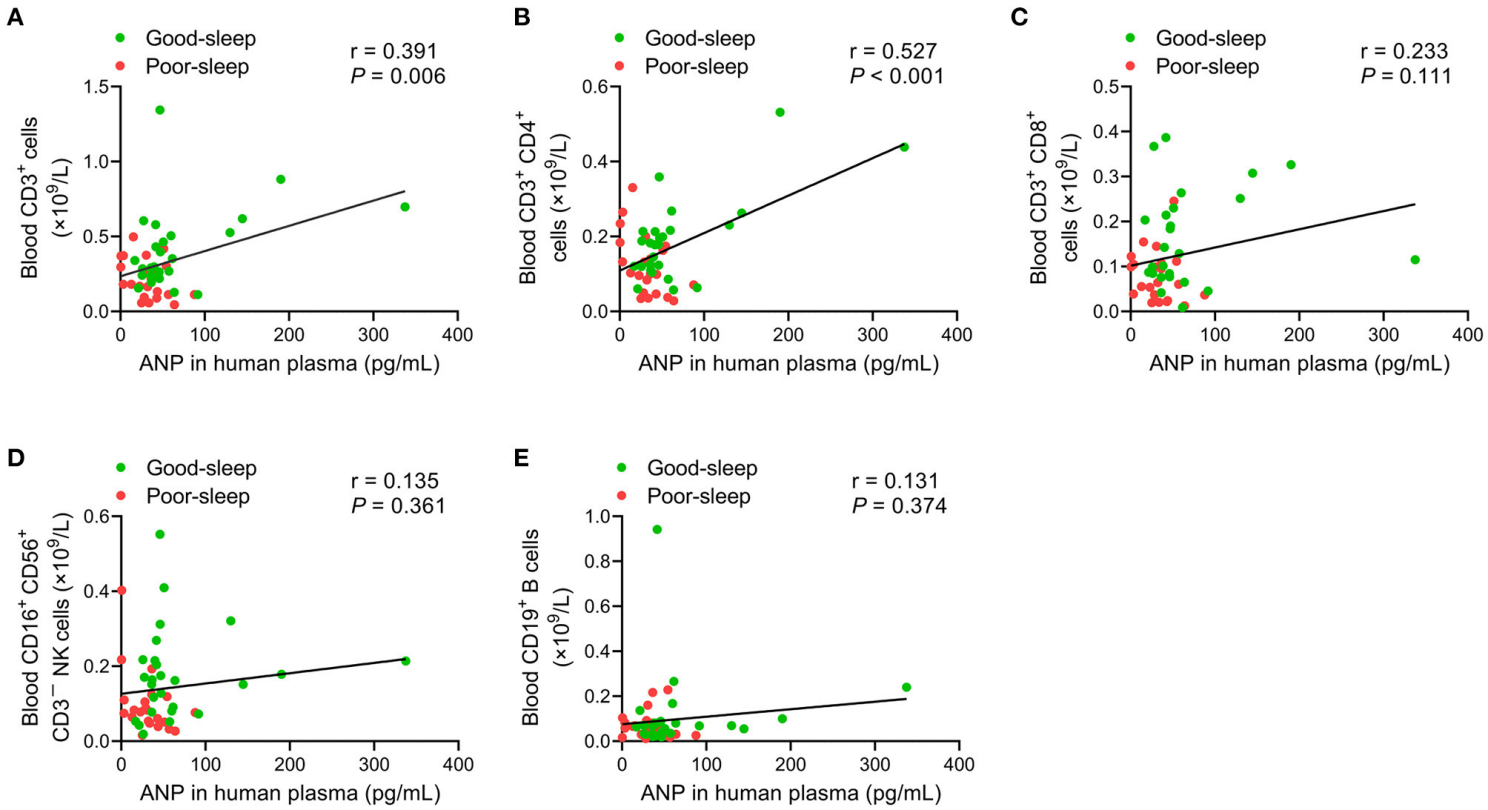
Correlation analysis of the plasma levels of atrial natriuretic peptide (ANP) with lymphocyte subsets in the peripheral blood. The plasma levels of ANP at 24 h after inclusion were positively correlated with the number of CD3^+^ T-lymphocytes (*r* = 0.391, *P* = 0.006) **(A)** or the number of CD3^+^ CD4^+^ T-lymphocytes (*r* = 0.527, *P* < 0.001) **(B)** on day 5 after inclusion. No significant correlations between the plasma levels of ANP at 24 h after inclusion with the number of CD3^+^ CD8^+^ T-lymphocytes **(C)**, the number of CD3^−^CD16^+^ CD56^+^ natural killer cells **(D)**, or the number of CD19^+^ B-lymphocytes **(E)** on day 5 after inclusion were detected.

An analysis of the correlation between plasma ANP levels at 24 h after inclusion and ALC on day 5 after inclusion did not show any correlation (all *P* > 0.05; Table 4). However, there were positive correlations between plasma ANP levels at 24 h after inclusion and the number of CD3^+^ T-lymphocytes (*r* = 0.391, *P* = 0.006) or the number of CD3^+^ CD4^+^ T-lymphocytes (*r* = 0.527, *P* < 0.001) in the peripheral blood on day 5 after inclusion (Figure 4).

## Discussion

Patients with sepsis caused by pathogenic infections, including bacteria and viruses, typically present lymphopenia (15, 33–36), which can serve as a biomarker for immunosuppression (37, 38). In addition, a continuous and sustained decrease in total circulating lymphocytes is closely associated with disease aggravation and death in septic patients (33, 39). Through prospectively comparing the effects of very-short-term self-reported good or poor sleep on ALC and the recovery rate of ALC over the subsequent 1–3 days, we found that 2 consecutive days of poor sleep decreased ALC and dampened ALC recovery on day 5 after inclusion. Interestingly, there is a decreasing tendency for these two parameters on days 3 and 4 in septic patients with 2 consecutive days of poor sleep compared to septic patients with good sleep. Our data indicated that very-short-term poor sleep could worsen post-septic immunosuppression as demonstrated by a significant difference in ALC on day 5 between the two groups, although the sleep quality of the patients in the poor sleep group was improved in the following three nights. Furthermore, our previous study found that recovery rate based on ALC after sepsis could also reflect recovery from lymphopenia; 2–3 weeks of self-reported poor sleep quality during hospitalization in COVID-19 patients with lymphopenia was associated with a slow recovery of ALC (15). In our present study, very-short-term poor sleep in septic patients could slow down ALC recovery on day 3 after 2 consecutive days of poor sleep, which indicated that very-short-term poor sleep could not only decrease ALC but could also dampen ALC recovery on day 3 after 2 consecutive days of poor sleep in septic ICU patients. Our results suggest that sleep deprivation, even very-short-term sleep deprivation, could exert important detrimental effects on the recovery rate of post-septic immunosuppression. However, the reason why there was a delay between the 2 consecutive days of poor sleep and decreased ALC or slower ALC recovery is unclear, which may be because the immune function needs a certain amount of time to be suppressed. The sepsis-induced immunosuppression peaks several days after sepsis; immunostimulation therapy could promote the recovery of immune function and improve sepsis mortality (40, 41). Interestingly, the poor sleep patients had lower blood albumin levels compared to the good sleep patients, suggesting that the relatively lower levels of blood albumin in poor sleep patients may affect subsequent ALC recovery to some extent due to the potential immunomodulatory and anti-inflammatory effects of albumin in critically ill patients (42–44). Therapeutic preparations of human albumin are shown to be able to promote the major histocompatibility complex class II-restricted activation of antigen-specific T cells (43). Albumin preconditioning could abrogate the lipopolysaccharides (LPS)-mediated increase in the protein levels of tumor necrosis factor alpha (TNF-α) in cultured macrophages and septic mice (44). Due to the negative correlation between hyperinflammation and systemic immune function (38, 45, 46), hyperinflammation mediated by reduced levels of albumin in the poor sleep group would also compromise the immune function.

ANP, a cardiovascular hormone mainly secreted by the heart atria (47), plays important protective roles in many diseases, including ventricular hypertrophy, myocardial injury, atherosclerosis, hypertension, tumor, acute lung injury, ischemia/reperfusion injury, and sepsis (48, 49). ANP has immunomodulatory capacity to stimulate the differentiation of naive CD4^+^ cells toward the T helper (Th) 2 and/or Th17 phenotype (48). In addition to regulating adaptive immunity, ANP is also involved in innate immunity, being able to stimulate macrophage phagocytosis, promote reactive oxygen species release, increase NK cytotoxicity, inhibit the synthesis and release of proinflammatory mediators (TNF-α, interleukin-1, monocyte chemoattractant protein 1, nitric oxide, cyclooxygenase-2, *etc*.), and reduce the expression of adhesion molecules (vascular cell adhesion molecules, intercellular cell adhesion molecule-1, E-selectin, *etc*.) (48, 50–53). The anti-inflammatory and immunomodulatory effects of ANP may be through the activation of guanylyl cyclase-coupled receptor A (GC-A)/cyclic guanosine monophosphate (cGMP) signaling in dendritic cells (48). In an experimental model of sepsis, pretreatment of mice with ANP resulted in improved survival of mice after LPS challenge (54), which may be due to the potent inhibitory effect of ANP on LPS and TNF-α-induced increase in endothelial cell permeability (49). One previous study showed that chronic sleep deprivation for 1 month could abrogate an exercise-induced increase in the plasma levels of ANP, without obvious effects on the plasma levels of BNP (55). Consistently, in our present study, we found that 2 consecutive days of poor sleep could markedly decreased the plasma ANP levels, without significant effects on plasma BNP levels. Surprisingly, the plasma ANP levels at 24 h after inclusion were significantly positively correlated to ALC recovery on day 5 after inclusion, indicating that ANP might play important roles in sleep-mediated immunoregulation. The promoting effects of ANP on the early recovery of immune function may be due to its anti-inflammatory effects (48–54, 56) because hyperinflammation is negatively associated with systemic immune function (38, 45, 46). In addition, ANP can also affect the adaptive immunity, being able to stimulate the differentiation of naïve CD4^+^ cells (48) and promote dendritic cell-mediated T cell polarization (57) mediated by its principal receptor GC-A, which is highly expressed in thymus (58) and immune cells (including macrophages, dendritic cells, and T lymphocytes) (59). Our data also indicated that the plasma level of ANP could be used as a predictor of recovery of immune function in septic patients. However, no significant correlation between the plasma ANP levels at 24 h after inclusion and ALC on day 5 after inclusion was observed in our study, which remains unknown.

The RCSQ, a potential practical and valid instrument with feasibility and low cost, has been the most widely used subjective survey instrument for measuring the sleep quality of ICU patients (30–32). In a clinical study of 70 ICU patients, the RCSQ had been validated against polysomnography (32), which is considered as the gold-standard method for evaluating sleep. We lowered the upper limit of the RCSQ score that defines poor sleep quality (≤25) to minimize bias, while the cutoff point differentiating good and poor sleep was 70/100 in previous studies (60, 61). In addition, after the patients' self-reported sleep assessment, we further checked by asking the night-shift nurses who completed the RCSQ regarding their patients' overnight sleep quality to minimize the recollection and response bias caused by subjective assessment. Nurses may overestimate the patients' perceived sleep quality on the RCSQ compared with their patients (62); thus, we used patient–nurse interraters to ensure the patients' poor sleep. Because the appropriateness of the patients' self-reported sleep quality as assessed by RCSQ would be compromised if they are sedated and/or delirious, the patients with deep sedation or delirium during the study period were excluded in our study.

In the included patients with sepsis, the most important etiological causes of poor sleep were environmental and psychological factors, which accounted for 83.3 and 62.5%, respectively. Another major factor that contributed to the patients' poor sleep is physical discomfort caused by an illness. These factors are the major causal factors of sleep disturbance in ICU patients and could negatively affect their recovery from a critical illness (63–67). Therefore, improving the ward environment (reducing the light and noise levels at night, segregating patients from each other by curtains, using earplugs or eye masks, *etc*.), psychological therapy [social support intervention, spiritual encouragement, psychological comfort, emotional support, relaxation intervention plus relaxing music (muscle relaxation, mental imagery, audiotape), psychotropic drug therapy, *etc*.], and alleviating psychological comfort (adequate analgesia, proper sedation, optimizing ventilator mode or type, *etc*.) are essential to improve the sleep quality of septic patients in ICU wards (68). Furthermore, we found that the patients with poor sleep received longer days of sedation before inclusion, indicating that long-term sedation may be associated with poor sleep afterwards. In our study, the invasive mechanically ventilated patients were primarily sedated with propofol before and after inclusion. Propofol has been shown to have a negative immunomodulatory effect (69–72); it can decrease T-cell proliferation and IL-2 production (71). Therefore, patients with poor sleep in our study had a longer period of sedation before inclusion, which may also lead to a subsequent slower recovery of immune function.

There were some notable limitations in our study. First, it was a single-center study with a small sample size. Future prospective studies with large patient cohorts are needed to validate the results. Second, although multiple criteria for grouping based on the RCSQ were strictly defined in this study, there could still be a recollection and response bias. Therefore, objective sleep monitoring is needed in future studies. Third, bidirectional causality might exist between poor sleep quality and the recovery of immune function in septic patients. We cannot rule out the possibility that the underlying more severe condition caused the poor sleep quality in septic patients, although no significant between-group differences in the APACHE II score and SOFA score as well as laboratory parameters were detected within 24 h of inclusion. Fourth, the sleep quality in the ICU before inclusion could not be assessed due to the sepsis-induced brain dysfunction and administration of deep sedation. Whether possible differences in circadian rhythm changes before inclusion between the two groups could have an impact on the subsequent recovery of immune function after sepsis is unclear. Fifth, a relatively small group of ICU patients who were awake and able to communicate were included in our study; thus, selecting only a subgroup of less severely ill patients could make it difficult to generalize our findings to the whole ICU population. Although sleep quality assessment in patients with sedation and/or delirium remains difficult, interventions aimed at improving sleep quality may also have beneficial effects. Sixth, the long-term impact of very-short-term sleep deprivation on patient outcomes was not taken into account. The very-short-term sleep deprivation may exert long-term negative consequences in the recovery of immune function in septic patients.

In conclusion, in septic patients who were recovering from critical illness, very-short-term poor sleep quality was associated with a slow recovery of lymphocytes over the following few days, and the plasma levels of ANP were positively correlated with the subsequent recovery of lymphocytes. Therefore, continuous sleep deprivation or several short-term sleep deprivations with short intervals may continually worsen the recovery of immune function during recovery from critical illness. Poor recovery of immune function could negatively affect the prognosis of septic patients (33, 39). Attention should be paid to the patient's sleep quality every day. It is important to adopt comprehensive treatment measures during hospitalization to improve the sleep quality of septic patients to promote the recovery of immune function.

## Data Availability Statement

The raw data supporting the conclusions of this article will be made available by the authors, without undue reservation.

## Ethics Statement

The study was approved by the Ethics Committee of Union Hospital, Tongji Medical College, Huazhong University of Science and Technology (Permission number: S1164). The patients/participants provided their written informed consent to participate in this study.

## Author Contributions

JZ and SY had full access to all data in the study and take responsibility for the integrity of the data and the accuracy of the data analysis and contributed to the concept and design. JZ, YZ, YW, DX, PX, BX, and HH contributed to the acquisition, analysis, or interpretation of data. JZ contributed to the drafting of the manuscript and the statistical analysis. SY contributed to the critical revision of the manuscript for important intellectual content and supervised the study. YS and SY provided administrative, technical, or material support. All authors contributed to the article and approved the submitted version.

## Conflict of Interest

The authors declare that the research was conducted in the absence of any commercial or financial relationships that could be construed as a potential conflict of interest.

## References

[1] Besedovsky L Lange T Haack M. The sleep-immune crosstalk in health and disease. Physiol Rev. (2019) 99:1325–80. 10.1152/physrev.00010.2018 30920354 PMC6689741

[2] Haspel JA Anafi R Brown MK Cermakian N Depner C Desplats P. Perfect timing: circadian rhythms, sleep, and immunity - an NIH workshop summary. JCI Insight. (2020) 5:e131487. 10.1172/jci.insight.131487 31941836 PMC7030790

[3] Mukherjee S Patel SR Kales SN Ayas NT Strohl KP Gozal D. An official american thoracic society statement: the importance of healthy sleep. Recommendations and future priorities. Am J Respir Crit Care Med. (2015) 191:1450–8. 10.1164/rccm.201504-0767ST 26075423 PMC5442970

[4] Irwin MR. Why sleep is important for health: a psychoneuroimmunology perspective. Annu Rev Psychol. (2015) 66:143–72. 10.1146/annurev-psych-010213-115205 25061767 PMC4961463

[5] Haack M Sanchez E Mullington JM. Elevated inflammatory markers in response to prolonged sleep restriction are associated with increased pain experience in healthy volunteers. Sleep. (2007) 30:1145–52. 10.1093/sleep/30.9.1145 17910386 PMC1978405

[6] Vgontzas AN Zoumakis E Bixler EO Lin HM Follett H Kales A. Adverse effects of modest sleep restriction on sleepiness, performance, and inflammatory cytokines. J Clin Endocrinol Metab. (2004) 89:2119–26. 10.1210/jc.2003-031562 15126529

[7] Bollinger T Bollinger A Skrum L Dimitrov S Lange T Solbach W. Sleep–dependent activity of T cells and regulatory T cells. Clin Exp Immunol. (2009) 155:231–8. 10.1111/j.1365-2249.2008.03822.x 19040608 PMC2675254

[8] Toth LA Krueger JM. Alteration of sleep in rabbits by *Staphylococcus aureus* infection. Infect Immun. (1988) 56:1785–91. 10.1128/IAI.56.7.1785-1791.1988 3384477 PMC259478

[9] Cohen S Doyle WJ Alper CM Janicki-Deverts D Turner RB. Sleep habits and susceptibility to the common cold. Arch Intern Med. (2009) 169:62–7. 10.1001/archinternmed.2008.505 19139325 PMC2629403

[10] Prather AA Janicki-Deverts D Hall MH Cohen S. Behaviorally assessed sleep and susceptibility to the common cold. Sleep. (2015) 38:1353–9. 10.5665/sleep.4968 26118561 PMC4531403

[11] Patel SR Malhotra A Gao X Hu FB Neuman MI Fawzi WW. A prospective study of sleep duration and pneumonia risk in women. Sleep. (2012) 35:97–101. 10.5665/sleep.1594 22215923 PMC3242694

[12] Loef B van Baarle D van der Beek AJ Sanders E Bruijning-Verhagen P Proper KI. Shift work and respiratory infections in health-care workers. Am J Epidemiol. (2019) 188:509–17. 10.1093/aje/kwy258 30475977 PMC6395171

[13] Kuo TH Williams JA. Increased sleep promotes survival during a bacterial infection in Drosophila. Sleep. (2014) 37:1077–86. 10.5665/sleep.3764 24882902 PMC4015381

[14] Preston BT Capellini I McNamara P Barton RA Nunn CL. Parasite resistance and the adaptive significance of sleep. BMC Evol Biol. (2009) 9:7. 10.1186/1471-2148-9-7 19134175 PMC2631508

[15] Zhang J Xu D Xie B Zhang Y Huang H Liu H. Poor-sleep is associated with slow recovery from lymphopenia and an increased need for ICU care in hospitalized patients with COVID-19: a retrospective cohort study. Brain Behav Immun. (2020) 88:50–8. 10.1016/j.bbi.2020.05.075 32512133 PMC7274970

[16] Gabor JY Cooper AB Hanly PJ. Sleep disruption in the intensive care unit. Curr Opin Crit Care. (2001) 7:21–7. 10.1097/00075198-200102000-00004 11373507

[17] Pisani MA Friese RS Gehlbach BK Schwab RJ Weinhouse GL Jones SF. Sleep in the intensive care unit. Am J Respir Crit Care Med. (2015) 191:731–8. 10.1164/rccm.201411-2099CI 25594808 PMC5447310

[18] Cecconi M Evans L Levy M Rhodes A. Sepsis and septic shock. Lancet. (2018) 392:75–87. 10.1016/S0140-6736(18)30696-2 29937192

[19] Krueger JM Majde JA. Cytokines and sleep. Int Arch Allergy Immunol. (1995) 106:97–100. 10.1159/000236827 7819749

[20] Majde JA Krueger JM. Links between the innate immune system and sleep. J Allergy Clin Immunol. (2005) 116:1188–98. 10.1016/j.jaci.2005.08.005 16337444

[21] Drake CL Roehrs TA Royer H Koshorek G Turner RB Roth T. Effects of an experimentally induced rhinovirus cold on sleep, performance, and daytime alertness. Physiol Behav. (2000) 71:75–81. 10.1016/S0031-9384(00)00322-X 11134688 PMC7134541

[22] Sharpley AL Cooper CM Williams C Godlewska BR Cowen PJ. Effects of typhoid vaccine on inflammation and sleep in healthy participants: a double-blind, placebo-controlled, crossover study. Psychopharmacology. (2016) 233:3429–35. 10.1007/s00213-016-4381-z 27503474 PMC4989013

[23] Huang CY Chen YT Wu LA Liu CJ Chang SC Perng DW. Sleep apnoea patients have higher mortality when confronting sepsis. Eur J Clin Invest. (2014) 44:38–45. 10.1111/eci.12187 24117403

[24] Friese RS Bruns B Sinton CM. Sleep deprivation after septic insult increases mortality independent of age. J Trauma. (2009) 66:50–4. 10.1097/TA.0b013e318190c3a1 19131805

[25] Fondell E Axelsson J Franck K Ploner A Lekander M Balter K. Short natural sleep is associated with higher T cell and lower NK cell activities. Brain Behav Immun. (2011) 25:1367–75. 10.1016/j.bbi.2011.04.004 21496482

[26] Irwin M Mascovich A Gillin JC Willoughby R Pike J Smith TL. Partial sleep deprivation reduces natural killer cell activity in humans. Psychosom Med. (1994) 56:493–8. 10.1097/00006842-199411000-00004 7871104

[27] Irwin M McClintick J Costlow C Fortner M White J Gillin JC. Partial night sleep deprivation reduces natural killer and cellular immune responses in humans. FASEB J. (1996) 10:643–53. 10.1096/fasebj.10.5.8621064 8621064

[28] De Lorenzo BH de Oliveira ML Greco CR Suchecki D. Sleep-deprivation reduces NK cell number and function mediated by beta-adrenergic signalling. Psychoneuroendocrinology. (2015) 57:134–43. 10.1016/j.psyneuen.2015.04.006 25929826

[29] Wilder-Smith A Mustafa FB Earnest A Gen L Macary PA. Impact of partial sleep deprivation on immune markers. Sleep Med. (2013) 14:1031–4. 10.1016/j.sleep.2013.07.001 23993876

[30] Nagatomo K Masuyama T Iizuka Y Makino J Shiotsuka J Sanui M. Validity of an under-mattress sensor for objective sleep measurement in critically ill patients: a prospective observational study. J Intensive Care. (2020) 8:16. 10.1186/s40560-020-0433-x 32071722 PMC7014714

[31] Simons KS Verweij E Lemmens P Jelfs S Park M Spronk PE. Noise in the intensive care unit and its influence on sleep quality: a multicenter observational study in Dutch intensive care units. Crit Care. (2018) 22:250. 10.1186/s13054-018-2182-y 30290829 PMC6173893

[32] Richards KC O'Sullivan PS Phillips RL. Measurement of sleep in critically ill patients. J Nurs Meas. (2000) 8:131–44. 10.1891/1061-3749.8.2.131 11227580

[33] Wang D Hu B Hu C Zhu F Liu X Zhang J. Clinical characteristics of 138 hospitalized patients with 2019 novel coronavirus-infected pneumonia in Wuhan, China. JAMA. (2020) 323:1061–9. 10.1001/jama.2020.1585 32031570 PMC7042881

[34] Huang C Wang Y Li X Ren L Zhao J Hu Y. Clinical features of patients infected with 2019 novel coronavirus in Wuhan, China. Lancet. (2020) 395:497–506. 10.1016/S0140-6736(20)30183-5 31986264 PMC7159299

[35] Drewry AM Fuller BM Skrupky LP Hotchkiss RS. The presence of hypothermia within 24 hours of sepsis diagnosis predicts persistent lymphopenia. Crit Care Med. (2015) 43:1165–9. 10.1097/CCM.0000000000000940 25793436 PMC4700928

[36] Carvelli J Piperoglou C Bourenne J Farnarier C Banzet N Demerle C. Imbalance of circulating innate lymphoid cell subpopulations in patients with septic shock. Front Immunol. (2019) 10:2179. 10.3389/fimmu.2019.02179 31616411 PMC6763762

[37] Drewry AM Samra N Skrupky LP Fuller BM Compton SM Hotchkiss RS. Persistent lymphopenia after diagnosis of sepsis predicts mortality. Shock. (2014) 42:383–91. 10.1097/SHK.0000000000000234 25051284 PMC4362626

[38] Chen X Yuan S Zhang J. Correlation study between blood cytokines and lymphocytes in early postoperative critical patients with compromised immune function. Medicine. (2020) 99:e22459. 10.1097/MD.0000000000022459 33080681 PMC7571877

[39] Zhou F Yu T Du R Fan G Liu Y Liu Z. Clinical course and risk factors for mortality of adult inpatients with COVID-19 in Wuhan, China: a retrospective cohort study. Lancet. (2020) 395:1054–62. 10.1016/S0140-6736(20)30566-3 32171076 PMC7270627

[40] Hotchkiss RS Monneret G Payen D. Immunosuppression in sepsis: a novel understanding of the disorder and a new therapeutic approach. Lancet Infect Dis. (2013) 13:260–8. 10.1016/S1473-3099(13)70001-X 23427891 PMC3798159

[41] Hotchkiss RS Monneret G Payen D. Sepsis-induced immunosuppression: from cellular dysfunctions to immunotherapy. Nat Rev Immunol. (2013) 13:862–862 10.1038/nri3552 24232462 PMC4077177

[42] Ferrer R Mateu X Maseda E Yébenes JC Aldecoa C De Haro C. Non-oncotic properties of albumin. A multidisciplinary vision about the implications for critically ill patients. Expert Rev Clin Pharmacol. (2018) 11:125–37. 10.1080/17512433.2018.1412827 29219627

[43] Aubin E Roberge C Lemieux R Bazin R. Immunomodulatory effects of therapeutic preparations of human albumin. Vox Sang. (2011) 101:131–13 10.1111/j.1423-0410.2011.01475.x 21426357

[44] Wheeler DS Giuliano JS Jr Lahni PM Denenberg A Wong HR Zingarelli B. The immunomodulatory effects of albumin *in vitro* and *in vivo*. Adv Pharmacol Sci. (2011) 2011:691928. 10.1155/2011/691928 21603190 PMC3096151

[45] Giamarellos-Bourboulis EJ Netea MG Rovina N Akinosoglou K Antoniadou A Antonakos N. Complex immune dysregulation in COVID-19 patients with severe respiratory failure. Cell Host Microbe. (2020) 27:992–1000.e3. 10.1016/j.chom.2020.04.009 32320677 PMC7172841

[46] Wherry EJ Kurachi M. Molecular and cellular insights into T cell exhaustion. Nat Rev Immunol. (2015) 15:486–99. 10.1038/nri3862 26205583 PMC4889009

[47] Vesely DL. Metabolic targets of cardiac hormones' therapeutic anti-cancer effects. Curr Pharm Des. (2010) 16:1159–66. 10.2174/138161210790963887 20030620

[48] De Vito P. Atrial natriuretic peptide: an old hormone or a new cytokine? Peptides. (2014) 58:108–16. 10.1016/j.peptides.2014.06.011 24973596

[49] Xing J Birukova AA. ANP attenuates inflammatory signaling and Rho pathway of lung endothelial permeability induced by LPS and TNFalpha. Microvasc Res. (2010) 79:56–62. 10.1016/j.mvr.2009.11.006 19931545 PMC2813389

[50] Kiemer AK Hartung T Vollmar AM. cGMP-mediated inhibition of TNF-alpha production by the atrial natriuretic peptide in murine macrophages. J Immunol. (2000) 165:175–81. 10.4049/jimmunol.165.1.175 10861050

[51] Tsukagoshi H Shimizu Y Kawata T Hisada T Shimizu Y Iwamae S. Atrial natriuretic peptide inhibits tumor necrosis factor-alpha production by interferon-gamma-activated macrophages via suppression of p38 mitogen-activated protein kinase and nuclear factor-kappa B activation. Regul Pept. (2001) 99:21–9. 10.1016/S0167-0115(01)00218-X 11257311

[52] Weber NC Blumenthal SB Hartung T Vollmar AM Kiemer AK. ANP inhibits TNF-alpha-induced endothelial MCP-1 expression–involvement of p38 MAPK and MKP-1. J Leukoc Biol. (2003) 74:932–41. 10.1189/jlb.0603254 12960255

[53] Kiemer AK Vollmar AM. Autocrine regulation of inducible nitric-oxide synthase in macrophages by atrial natriuretic peptide. J Biol Chem. (1998) 273:13444–51. 10.1074/jbc.273.22.13444 9593677

[54] Ladetzki-Baehs K Keller M Kiemer AK Koch E Zahler S Wendel A. Atrial natriuretic peptide, a regulator of nuclear factor-kappaB activation *in vivo*. Endocrinology. (2007) 148:332–6. 10.1210/en.2006-0935 17008392

[55] Tanabe K Yamamoto A Suzuki N Akashi Y Seki A Samejima H. Exercise-induced changes in plasma atrial natriuretic peptide and brain natriuretic peptide concentrations in healthy subjects with chronic sleep deprivation. Jpn Circ J. (1999) 63:447–52. 10.1253/jcj.63.447 10406584

[56] Staedtke V Bai RY Kim K Darvas M Davila ML Riggins GJ. Disruption of a self-amplifying catecholamine loop reduces cytokine release syndrome. Nature. (2018) 564:273–7. 10.1038/s41586-018-0774-y 30542164 PMC6512810

[57] Morita R Ukyo N Furuya M Uchiyama T Hori T. Atrial natriuretic peptide polarizes human dendritic cells toward a Th2-promoting phenotype through its receptor guanylyl cyclase-coupled receptor A. J Immunol. (2003) 170:5869–75. 10.4049/jimmunol.170.12.5869 12794112

[58] Vollmar AM Schmidt KN Schulz R. Natriuretic peptide receptors on rat thymocytes: inhibition of proliferation by atrial natriuretic peptide. Endocrinology. (1996) 137:1706–13. 10.1210/endo.137.5.8612505 8612505

[59] Mohapatra SS Lockey RF Vesely DL Gower WR Jr. Natriuretic peptides and genesis of asthma: an emerging paradigm? J Allergy Clin Immunol. (2004) 114:520–6. 10.1016/j.jaci.2004.05.028 15356551

[60] Mannion H Molloy DW O'Caoimh R. Sleep disturbance in older patients in the emergency department: prevalence, predictors and associated outcomes. Int J Environ Res Public Health. (2019) 16:3577. 10.3390/ijerph16193577 31557801 PMC6801409

[61] McKinley S Fien M Elliott R Elliott D. Sleep and psychological health during early recovery from critical illness: an observational study. J Psychosom Res. (2013) 75:539–45. 10.1016/j.jpsychores.2013.09.007 24290043

[62] Kamdar BB Shah PA King LM Kho ME Zhou X Colantuoni E. Patient-nurse interrater reliability and agreement of the Richards-Campbell sleep questionnaire. Am J Crit Care. (2012) 21:261–9. 10.4037/ajcc2012111 22751369 PMC3667655

[63] Delaney LJ Van Haren F Lopez V. Sleeping on a problem: the impact of sleep disturbance on intensive care patients - a clinical review. Ann Intensive Care. (2015) 5:3. 10.1186/s13613-015-0043-2 25852963 PMC4385145

[64] Devlin JW Skrobik Y Gelinas C Needham DM Slooter A Pandharipande PP. Clinical practice guidelines for the prevention and management of pain, agitation/sedation, delirium, immobility, and sleep disruption in adult patients in the ICU. Crit Care Med. (2018) 46:e825–73. 10.1097/CCM.0000000000003259 30113379

[65] Pulak LM Jensen L. Sleep in the intensive care unit: a review. J Intensive Care Med. (2016) 31:14–23. 10.1177/0885066614538749 24916753

[66] Kamdar BB Needham DM Collop NA. Sleep deprivation in critical illness: its role in physical and psychological recovery. J Intensive Care Med. (2012) 27:97–111. 10.1177/0885066610394322 21220271 PMC3299928

[67] Friese RS. Sleep and recovery from critical illness and injury: a review of theory, current practice, and future directions. Crit Care Med. (2008) 36:697–705. 10.1097/CCM.0B013E3181643F29 18176314

[68] Hu RF Jiang XY Chen J Zeng Z Chen XY Li Y. Non-pharmacological interventions for sleep promotion in the intensive care unit. Cochrane Database Syst Rev. (2015) 2015:D8808. 10.1002/14651858.CD008808.pub2 26439374 PMC6517220

[69] Visvabharathy L Freitag NE. Propofol sedation exacerbates kidney pathology and dissemination of bacteria during *Staphylococcus aureus* bloodstream infections. Infect Immun. (2017) 85:e00097–17. 10.1128/IAI.00097-17 28461390 PMC5478955

[70] Chen MS Lin WC Yeh HT Hu CL Sheu SM. Propofol specifically suppresses IL-1β secretion but increases bacterial survival in *Staphylococcus aureus*-infected RAW264.7 cells. Mol Cell Biochem. (2018) 449:117–25. 10.1007/s11010-018-3348-2 29667111 PMC6223810

[71] Yuki K Soriano SG Shimaoka M. Sedative drug modulates T-cell and lymphocyte function-associated antigen-1 function. Anesth Analg. (2011) 112:830–8. 10.1213/ANE.0b013e31820dcabb 21385989 PMC3073815

[72] Visvabharathy L Xayarath B Weinberg G Shilling RA Freitag NE. Propofol increases host susceptibility to microbial infection by reducing subpopulations of mature immune effector cells at sites of infection. PLoS ONE. (2015) 10:e0138043. 10.1371/journal.pone.0138043 26381144 PMC4575148

